# Supplementary material for: Implications of the prevalence and magnitude of sustained declines for determining a minimum threshold for favourable population size
Source: PLoS One. 2020 Feb 12;15(2):e0228742. doi: 10.1371/journal.pone.0228742 (PMC7015407; doi:10.1371/journal.pone.0228742)
Supplement: S1 Table — Columns show: the total length in years of the monitoring data for each species; the first and last years of total monitoring period and the original dataset sources are given. Source codes are: BoCC - smoothed trends fitted to annual data from the British Trust for Ornithology (BTO and Joint Nature Conservation Committee (JNCC) Common Birds Census (CBC) and the BTO/JNCC/Royal Society for the Protection of Birds (RSPB) Breeding Bird Survey (BBS) (Harris et al. 2015); RBBP - data from the Rare Breeding Birds Panel (Holling 2014); SCARRABS - national population estimates from surveys of single species, which were typically undertaken at intervals of at least several years (Eaton et al. 2015); Declines of at least 10 years duration were identified, and the first (t1) and last (t2) years of the declines are given. The population size or indexed population sizes n1 at t1 and n2 at t2 are given. References are as supplied in the main text. (DOCX) [file pone.0228742.s001.docx]

**Supporting information**

**S1 Table.** **Data on population changes of UK breeding bird species with more than 10 years of monitoring data available between 1970 and 2013.** Columns show: the total length in years of the monitoring data for each species; the first and last years of total monitoring period and the original dataset sources are given. Source codes are: BoCC - smoothed trends fitted to annual data from the British Trust for Ornithology (BTO and Joint Nature Conservation Committee (JNCC) Common Birds Census (CBC) and the BTO/JNCC/Royal Society for the Protection of Birds (RSPB) Breeding Bird Survey (BBS) (Harris et al. 2015); RBBP - data from the Rare Breeding Birds Panel (Holling 2014); SCARRABS - national population estimates from surveys of single species, which were typically undertaken at intervals of at least several years (Eaton et al. 2015); Declines of at least 10 years duration were identified, and the first (t_1_) and last (t_2_) years of the declines are given. The population size or indexed population sizes n_1_ at t_1_  and n_2_ at t_2_ are given. References are as supplied in the main text.

| Common name | Species name | Length of time series | Source of data set | First year of time series | Last year of time series | First year of decline t_1_ | Population size/index at start n_1_ | Last year of decline t_2_ | Population size/index at end n_2_ |
| --- | --- | --- | --- | --- | --- | --- | --- | --- | --- |
| Arctic Skua | *Stercorarius parasiticus* | 43 | BoCC | 1970 | 2013 | 1986 | 3.11 | 2013 | 0.56 |
| Arctic Tern | *Sterna paradisaea* | 43 | BoCC | 1970 | 2013 | 1975 | 2.26 | 2004 | 1.29 |
| Avocet | *Recurvirostra avosetta* | 43 | RBBP | 1970 | 2013 |  |  |  |  |
| Bearded tit | *Panurus biarmicus* | 43 | RBBP | 1970 | 2013 | 1977 | 665 | 1991 | 242 |
| Bittern | *Botaurus stellaris* | 43 | RBBP | 1970 | 2013 | 1970 | 72 | 1997 | 11 |
| Black Grouse | *Tetrao tetrix* | 10 | SCARRABS | 1995 | 2005 | 1995 | 6506 | 2005 | 5078 |
| Black redstart | *Phoenicurus ochruros* | 43 | RBBP | 1970 | 2013 | 1986 | 114 | 2007 | 41 |
| Blackbird | *Turdus merula* | 43 | BoCC | 1970 | 2013 | 1970 | 1 | 1995 | 0.69 |
| Blackcap | *Sylvia atricapilla* | 43 | BoCC | 1970 | 2013 |  |  |  |  |
| Black-headed Gull | *Larus ridibundus* | 43 | BoCC | 1970 | 2013 | 1991 | 1.47 | 2003 | 0.99 |
| Black-necked grebe | *Podiceps nigricollis* | 43 | RBBP | 1970 | 2013 | 1994 | 81 | 2008 | 43 |
| Black-tailed godwit | *Limosa limosa* | 43 | RBBP | 1970 | 2013 | 1976 | 87 | 1993 | 33 |
| Black-throated Diver | *Gavia arctica* | 21 | SCARRABS | 1985 | 2006 |  |  |  |  |
| Black-winged stilt | *Himantopus himantopus* | 30 | RBBP | 1983 | 2013 |  |  |  |  |
| Blue Tit | *Cyanistes caeruleus* | 43 | BoCC | 1970 | 2013 |  |  |  |  |
| Bluethroat | *Luscinia svecica* | 33 | RBBP | 1980 | 2013 |  |  |  |  |
| Brambling | *Fringilla montifringilla* | 37 | RBBP | 1976 | 2013 |  |  |  |  |
| Bullfinch | *Pyrrhula pyrrhula* | 43 | BoCC | 1970 | 2013 | 1974 | 1.11 | 2000 | 0.46 |
| Buzzard | *Buteo buteo* | 43 | BoCC | 1970 | 2013 |  |  |  |  |
| Capercaillie | *Tetrao urogallus* | 43 | BoCC | 1970 | 2013 | 1970 | 1 | 2013 | 0.09 |
| Carrion Crow | *Corvus corone* | 43 | BoCC | 1970 | 2013 |  |  |  |  |
| Cetti's warbler | *Cettia cetti* | 43 | RBBP | 1970 | 2013 |  |  |  |  |
| Chaffinch | *Fringilla coelebs* | 43 | BoCC | 1970 | 2013 |  |  |  |  |
| Chiffchaff | *Phylloscopus collybita* | 43 | BoCC | 1970 | 2013 | 1970 | 1 | 1984 | 0.49 |
| Cirl bunting | *Emberiza cirlus* | 43 | RBBP | 1970 | 2013 |  |  |  |  |
| Coal Tit | *Periparus ater* | 43 | BoCC | 1970 | 2013 | 1975 | 1.28 | 1987 | 1.06 |
| Collared Dove | *Streptopelia decaocto* | 43 | BoCC | 1970 | 2013 |  |  |  |  |
| Common Crossbill | *Loxia curvirostra* | 19 | BoCC | 1994 | 2013 |  |  |  |  |
| Common Sandpiper | *Actitis hypoleucos* | 38 | BoCC | 1975 | 2013 | 1985 | 1.28 | 2013 | 0.57 |
| Common scoter | *Melanitta nigra* | 43 | RBBP | 1970 | 2013 | 1970 | 176 | 2006 | 16 |
| Common Tern | *Sterna hirundo* | 43 | BoCC | 1970 | 2013 |  |  |  |  |
| Coot | *Fulica atra* | 38 | BoCC | 1975 | 2013 |  |  |  |  |
| Cormorant | *Phalacrocorax carbo* | 43 | BoCC | 1970 | 2013 | 1995 | 1.51 | 2013 | 0.99 |
| Corn Bunting | *Miliaria calandra* | 43 | BoCC | 1970 | 2013 | 1973 | 1.09 | 2013 | 0.09 |
| Corncrake | *Crex crex* | 43 | BoCC | 1970 | 2013 | 1970 | 1 | 1992 | 0.19 |
| Crane | *Grus grus* | 43 | RBBP | 1970 | 2013 |  |  |  |  |
| Cuckoo | *Cuculus canorus* | 43 | BoCC | 1970 | 2013 | 1979 | 1.06 | 2013 | 0.38 |
| Curlew | *Numenius arquata* | 34 | BoCC | 1979 | 2013 | 1995 | 1.54 | 2013 | 0.88 |
| Dartford warbler | *Sylvia undata* | 43 | RBBP | 1970 | 2013 |  |  |  |  |
| Dipper | *Cinclus cinclus* | 38 | BoCC | 1975 | 2013 | 1975 | 1.08 | 2013 | 0.70 |
| Dunnock | *Prunella modularis* | 43 | BoCC | 1970 | 2013 |  |  |  |  |
| Eurasian Dotterel | *Charadrius morinellus* | 24 | SCARRABS | 1987 | 2011 | 1987 | 980 | 2011 | 423 |
| European Nightjar | *Caprimulgus europaeus* | 23 | SCARRABS | 1981 | 2004 |  |  |  |  |
| Fieldfare | *Turdus pilaris* | 43 | RBBP | 1970 | 2013 |  |  |  |  |
| Firecrest | *Regulas ignicapillus* | 43 | RBBP | 1970 | 2013 |  |  |  |  |
| Fulmar | *Fulmarus glacialis* | 43 | BoCC | 1970 | 2013 | 1996 | 2.24 | 2013 | 1.37 |
| Gadwall | *Anas strepera* | 19 | BoCC | 1994 | 2013 |  |  |  |  |
| Garden Warbler | *Sylvia borin* | 43 | BoCC | 1970 | 2013 | 1988 | 1.27 | 2013 | 0.94 |
| Garganey | *Anas querquedula* | 43 | RBBP | 1970 | 2013 |  |  |  |  |
| Goldcrest | *Regulus regulus* | 43 | BoCC | 1970 | 2013 | 1974 | 1.783 | 1992 | 0.548 |
| Golden Eagle | *Aquila chrysaetos* | 21 | SCARRABS | 1982 | 2003 |  |  |  |  |
| Golden oriole | *Oriolus oriolus* | 43 | RBBP | 1970 | 2013 | 1988 | 41 | 2013 | 2 |
| Golden Plover | *Pluvialis apricaria* | 19 | BoCC | 1994 | 2013 |  |  |  |  |
| Goldeneye | *Bucephala clangula* | 43 | RBBP | 1970 | 2013 |  |  |  |  |
| Goldfinch | *Carduelis carduelis* | 43 | BoCC | 1970 | 2013 |  |  |  |  |
| Goosander | *Mergus merganser* | 33 | BoCC | 1980 | 2013 |  |  |  |  |
| Goshawk | *Accipiter gentiles* | 43 | RBBP | 1970 | 2013 |  |  |  |  |
| Great Black-backed Gull | *Larus marinus* | 43 | BoCC | 1970 | 2013 | 1970 | 1 | 1991 | 0.88 |
| Great Black-backed Gull | *Larus marinus* | 43 | BoCC | 1970 | 2013 | 1999 | 1.099 | 2013 | 0.714 |
| Great Crested Grebe | *Podiceps cristatus* | 19 | BoCC | 1994 | 2013 |  |  |  |  |
| Great northern diver | *Gavia immer* | 43 | RBBP | 1970 | 2013 |  |  |  |  |
| Great Spotted Woodpecker | *Dendrocopos major* | 43 | BoCC | 1970 | 2013 |  |  |  |  |
| Great Tit | *Parus major* | 43 | BoCC | 1970 | 2013 |  |  |  |  |
| Green sandpiper | *Tringa ochropus* | 43 | RBBP | 1970 | 2013 |  |  |  |  |
| Green Woodpecker | *Picus viridis* | 43 | BoCC | 1970 | 2013 |  |  |  |  |
| Greenfinch | *Chloris chloris* | 43 | BoCC | 1970 | 2013 |  |  |  |  |
| Grey Heron | *Ardea cinerea* | 43 | BoCC | 1970 | 2013 |  |  |  |  |
| Grey Partridge | *Perdix perdix* | 43 | BoCC | 1970 | 2013 | 1971 | 1 | 2013 | 0.083 |
| Grey Wagtail | *Motacilla cinerea* | 38 | BoCC | 1975 | 2013 |  |  |  |  |
| Greylag Goose | *Anser anser* | 21 | BoCC | 1992 | 2013 |  |  |  |  |
| Guillemot | *Uria aalge* | 43 | BoCC | 1970 | 2013 |  |  |  |  |
| Hen Harrier | *Circus cyaneus* | 23 | BoCC | 1990 | 2013 |  |  |  |  |
| Herring Gull | *Larus argentatus* | 43 | BoCC | 1970 | 2013 | 1970 | 1 | 1991 | 0.395 |
| Herring Gull | *Larus argentatus* | 43 | BoCC | 1970 | 2013 | 1998 | 0.562 | 2010 | 0.368 |
| Hobby | *Falco subbuteo* | 43 | RBBP | 1970 | 2013 |  |  |  |  |
| Honey buzzard | *Pernis apivorus* | 43 | RBBP | 1970 | 2013 |  |  |  |  |
| Hooded Crow | *Corvus cornix* | 19 | BoCC | 1994 | 2013 |  |  |  |  |
| Hoopoe | *Upupa epops* | 43 | RBBP | 1970 | 2013 |  |  |  |  |
| House Martin | *Delichon urbicum* | 19 | BoCC | 1994 | 2013 |  |  |  |  |
| House Sparrow | *Passer domesticus* | 43 | BoCC | 1970 | 2013 | 1970 | 1 | 2007 | 0.701 |
| Icterine warbler | *Hippolais icterina* | 43 | RBBP | 1970 | 2013 |  |  |  |  |
| Jackdaw | *Coloeus monedula* | 43 | BoCC | 1970 | 2013 |  |  |  |  |
| Jay | *Garrulus glandarius* | 43 | BoCC | 1970 | 2013 | 1985 | 1.082 | 1997 | 0.886 |
| Kestrel | *Falco tinnunculus* | 43 | BoCC | 1970 | 2013 | 1977 | 1.27 | 2013 | 0.474 |
| Kingfisher | *Alcedo atthis* | 38 | BoCC | 1975 | 2013 |  |  |  |  |
| Kittiwake | *Rissa tridactyla* | 43 | BoCC | 1970 | 2013 | 1990 | 1.329 | 2013 | 0.383 |
| Lapwing | *Vanellus vanellus* | 43 | BoCC | 1970 | 2013 | 1979 | 1.136 | 2013 | 0.329 |
| Lesser Black-backed Gull | *Larus fuscus* | 43 | BoCC | 1970 | 2013 | 1997 | 2.182 | 2013 | 1.057 |
| Lesser Redpoll | *Carduelis caberet* | 43 | BoCC | 1970 | 2013 | 1975 | 1.256 | 1995 | 0.104 |
| Lesser Spotted Woodpecker | *Dendrocopos minor* | 43 | BoCC | 1970 | 2013 | 1979 | 1.84 | 2013 | 0.15 |
| Lesser Whitethroat | *Sylvia curruca* | 43 | BoCC | 1970 | 2013 | 1985 | 1.432 | 1998 | 0.898 |
| Linnet | *Linaria cannabina* | 43 | BoCC | 1970 | 2013 | 1975 | 1.08 | 1988 | 0.424 |
| Little Egret | *Egretta garzetta* | 7 | BoCC | 2006 | 2013 |  |  |  |  |
| Little Grebe | *Tachybaptus ruficollis* | 38 | BoCC | 1975 | 2013 |  |  |  |  |
| Little gull | *Hydrocoloeus minutus* | 43 | RBBP | 1970 | 2013 |  |  |  |  |
| Little Tern | *Sternula albifrons* | 43 | BoCC | 1970 | 2013 | 1976 | 1.169 | 2004 | 0.800 |
| Long-tailed Tit | *Aegithalos caudatus* | 43 | BoCC | 1970 | 2013 |  |  |  |  |
| Magpie | *Pica pica* | 43 | BoCC | 1970 | 2013 |  |  |  |  |
| Mallard | *Anas platyrhynchos* | 38 | BoCC | 1975 | 2013 |  |  |  |  |
| Marsh harrier | *Circus aeruginosus* | 43 | RBBP | 1970 | 2013 |  |  |  |  |
| Marsh Tit | *Parus palustris* | 43 | BoCC | 1970 | 2013 | 1970 | 1 | 2013 | 0.278 |
| Marsh warbler | *Acrocephalus palustris* | 43 | RBBP | 1970 | 2013 | 1970 | 91 | 2007 | 3 |
| Meadow pipit | *Anthus pratensis* | 43 | BoCC | 1970 | 2013 | 1975 | 1.27 | 2013 | 0.571 |
| Mediterranean gull | *Larus melanocephalus* | 36 | RBBP | 1976 | 2012 |  |  |  |  |
| Merlin | *Falco columbarius* | 25 | SCARRABS | 1983 | 2008 |  |  |  |  |
| Mistle Thrush | *Turdus viscivorus* | 43 | BoCC | 1970 | 2013 | 1976 | 1.04 | 2013 | 0.371 |
| Montagu's harrier | *Circus pygargus* | 43 | RBBP | 1970 | 2013 |  |  |  |  |
| Moorhen | *Gallinula chloropus* | 38 | BoCC | 1975 | 2013 | 1975 | 1.085 | 2013 | 0.688 |
| Mute Swan | *Cygnus olor* | 38 | BoCC | 1975 | 2013 |  |  |  |  |
| Nightingale | *Luscinia megarhynchos* | 19 | BoCC | 1994 | 2013 | 1994 | 0.919 | 2009 | 0.427 |
| Nuthatch | *Sitta europaea* | 43 | BoCC | 1970 | 2013 |  |  |  |  |
| Osprey | *Pandion haliaetus* | 43 | RBBP | 1970 | 2013 |  |  |  |  |
| Oystercatcher | *Haematopus ostralegus* | 38 | BoCC | 1975 | 2013 | 1995 | 2.4 | 2013 | 2.059 |
| Parrot crossbill | *Loxia pytyopsittacus* | 43 | RBBP | 1970 | 2013 |  |  |  |  |
| Peregrine | *Falco peregrinus* | 43 | BoCC | 1970 | 2013 |  |  |  |  |
| Pied Flycatcher | *Ficedula hypoleuca* | 19 | BoCC | 1994 | 2013 | 1995 | 0.93 | 2013 | 0.424 |
| Pied Wagtail | *Motacilla alba* | 43 | BoCC | 1970 | 2013 |  |  |  |  |
| Pintail | *Anas acuta* | 43 | RBBP | 1970 | 2013 |  |  |  |  |
| Pochard | *Aythya ferina* | 32 | RBBP | 1980 | 2012 |  |  |  |  |
| Purple sandpiper | *Calidris maritima* | 43 | RBBP | 1970 | 2013 |  |  |  |  |
| Quail | *Coturnix coturnix* | 33 | RBBP | 1979 | 2012 |  |  |  |  |
| Raven | *Corvus corax* | 19 | BoCC | 1994 | 2013 |  |  |  |  |
| Razorbill | *Alca torda* | 27 | BoCC | 1986 | 2013 |  |  |  |  |
| Red Grouse | *Lagopus lagopus* | 19 | BoCC | 1994 | 2013 |  |  |  |  |
| Red kite | *Milvus milvus* | 43 | RBBP | 1970 | 2013 |  |  |  |  |
| Red-backed shrike | *Lanius collurio* | 43 | RBBP | 1970 | 2013 | 1970 | 76 | 1995 | 1 |
| Red-billed Chough | *Pyrrhocorax pyrrhocorax* | 32 | SCARRABS | 1982 | 2014 |  |  |  |  |
| Red-breasted Merganser | *Mergus serrator* | 43 | BoCC | 1970 | 2013 | 1998 | 11.16 | 2010 | 6.919 |
| Red-necked grebe | *Podiceps grisegena* | 43 | RBBP | 1970 | 2013 |  |  |  |  |
| Red-necked phalarope | *Phalaropus lobatus* | 43 | RBBP | 1970 | 2013 |  |  |  |  |
| Redshank | *Tringa totanus* | 38 | BoCC | 1975 | 2013 | 1977 | 1.129 | 2013 | 0.419 |
| Redstart | *Phoenicurus phoenicurus* | 43 | BoCC | 1970 | 2013 |  |  |  |  |
| Red-throated Diver | *Gavia stellata* | 12 | SCARRABS | 1994 | 2006 |  |  |  |  |
| Redwing | *Turdus iliacus* | 43 | RBBP | 1970 | 2013 | 1979 | 79 | 2001 | 6 |
| Reed Bunting | *Emberiza schoeniclus* | 43 | BoCC | 1970 | 2013 |  |  |  |  |
| Reed Warbler | *Acrocephalus scirpaceus* | 33 | BoCC | 1980 | 2013 |  |  |  |  |
| Ring Ouzel | *Turdus torquatus* | 14 | SCARRABS | 1999 | 2013 | 1999 | 6853 | 2012 | 4996 |
| Robin | *Erithacus rubecula* | 43 | BoCC | 1970 | 2013 | 1972 | 1.046 | 1986 | 0.925 |
| Rook | *Corvus frugilegus* | 38 | BoCC | 1975 | 2013 | 2000 | 1.546 | 2013 | 1.168 |
| Roseate tern | *Sterna dougallii* | 43 | RBBP | 1970 | 2013 | 1971 | 1223 | 1997 | 50 |
| Ruff - females at leks | *Philomachus pugnax* | 43 | RBBP | 1970 | 2013 |  |  |  |  |
| Sand Martin | *Riparia riparia* | 36 | BoCC | 1977 | 2013 | 1989 | 1.594 | 2005 | 0.948 |
| Sandwich Tern | *Sterna sandvicensis* | 43 | BoCC | 1970 | 2013 |  |  |  |  |
| Savi's warbler | *Locustella luscinioides* | 43 | RBBP | 1970 | 2013 | 1979 | 30 | 1998 | 2 |
| Scarlet rosefinch | *Carpodacus erythrinus* | 43 | RBBP | 1970 | 2013 |  |  |  |  |
| Scaup | *Aythya marila* | 40 | RBBP | 1973 | 2013 |  |  |  |  |
| Sedge Warbler | *Acrocephalus schoenobaenus* | 38 | BoCC | 1975 | 2013 | 1975 | 1.085 | 2013 | 0.539 |
| Serin | *Serinus serinus* | 37 | RBBP | 1976 | 2013 |  |  |  |  |
| Shag | *Phalacrocorax aristotelis* | 43 | BoCC | 1970 | 2013 | 1989 | 1.52 | 2013 | 0.642 |
| Shelduck | *Tadorna tadorna* | 19 | BoCC | 1994 | 2013 |  |  |  |  |
| Shorelark | *Eremophila alpestris* | 43 | RBBP | 1970 | 2013 |  |  |  |  |
| Shoveler | *Anas clypeata* | 7 | BoCC | 2006 | 2013 |  |  |  |  |
| Siskin | *Spinus spinus* | 19 | BoCC | 1994 | 2013 |  |  |  |  |
| Skylark | *Alauda arvensis* | 43 | BoCC | 1970 | 2013 | 1976 | 1.09 | 2013 | 0.366 |
| Slavonian grebe | *Podiceps auritus* | 42 | RBBP | 1971 | 2013 | 1985 | 77 | 2010 | 22 |
| Snipe | *Gallinago gallinago* | 38 | BoCC | 1975 | 2013 | 1982 | 1.58 | 1996 | 0.176 |
| Snow bunting | *Plectrophenax nivalis* | 43 | RBBP | 1970 | 2013 |  |  |  |  |
| Song Thrush | *Turdus philomelos* | 43 | BoCC | 1970 | 2013 | 1970 | 0.99 | 1993 | 0.402 |
| Sparrowhawk | *Accipiter nisus* | 43 | BoCC | 1970 | 2013 |  |  |  |  |
| Spoonbill | *Platalea leucorodia* | 43 | RBBP | 1970 | 2013 |  |  |  |  |
| Spotted crake | *Porzana porzana* | 43 | RBBP | 1970 | 2013 |  |  |  |  |
| Spotted Flycatcher | *Muscisapa striata* | 43 | BoCC | 1970 | 2013 | 1971 | 0.99 | 2013 | 0.12 |
| Starling | *Sturna vulgaris* | 43 | BoCC | 1970 | 2013 | 1970 | 1 | 2013 | 0.185 |
| Stock Dove | *Columba oenas* | 43 | BoCC | 1970 | 2013 |  |  |  |  |
| Stone curlew | *Burhinus oedicnemus* | 43 | RBBP | 1970 | 2013 |  |  |  |  |
| Swallow | *Hirundo rustica* | 43 | BoCC | 1970 | 2013 |  |  |  |  |
| Swift | *Apus apus* | 19 | BoCC | 1994 | 2013 | 1994 | 0.92 | 2013 | 0.538 |
| Tawny Owl | *Strix aluco* | 43 | BoCC | 1970 | 2013 | 1972 | 1.057 | 2013 | 0.62 |
| Teal | *Anas crecca* | 19 | BoCC | 1994 | 2013 |  |  |  |  |
| Temminck's stint | *Calidris temminckii* | 43 | RBBP | 1970 | 2013 |  |  |  |  |
| Tree Pipit | *Anthus trivialis* | 43 | BoCC | 1970 | 2013 | 1970 | 1 | 2008 | 0.262 |
| Tree Sparrow | *Passer montanus* | 43 | BoCC | 1970 | 2013 | 1971 | 1.009 | 1997 | 0.043 |
| Treecreeper | *Certhia familiaris* | 43 | BoCC | 1970 | 2013 |  |  |  |  |
| Tufted Duck | *Aythya fuligula* | 38 | BoCC | 1975 | 2013 |  |  |  |  |
| Turtle Dove | *Streptopelia turtur* | 43 | BoCC | 1970 | 2013 | 1970 | 1 | 2013 | 0.031 |
| Twite | *Linaria flavirostris* | 14 | SCARRABS | 1999 | 2013 |  |  |  |  |
| Whinchat | *Saxicola rubetra* | 19 | BoCC | 1994 | 2013 | 1996 | 0.99 | 2012 | 0.429782 |
| White-tailed eagle | *Haliaeetus albicilla* | 43 | RBBP | 1970 | 2013 |  |  |  |  |
| Whitethroat | *Sylvia communis* | 43 | BoCC | 1970 | 2013 | 1970 | 0.733 | 1985 | 0.411 |
| Whooper swan | *Cygnus Cygnus* | 43 | RBBP | 1970 | 2013 |  |  |  |  |
| Willow Tit | *Parus montanus* | 43 | BoCC | 1970 | 2013 | 1973 | 1.03 | 2013 | 0.054 |
| Willow Warbler | *Phylloscopus trochilus* | 43 | BoCC | 1970 | 2013 | 1985 | 1.014 | 2005 | 0.544 |
| Wood Lark | *Lullula arborea* | 20 | SCARRABS | 1986 | 2006 |  |  |  |  |
| Wood sandpiper | *Tringa glareola* | 43 | RBBP | 1970 | 2013 |  |  |  |  |
| Wood Warbler | *Phylloscopus sibilatrix* | 19 | BoCC | 1994 | 2013 | 1994 | 0.89 | 2012 | 0.309 |
| Woodlark | *Lullula arborea* | 43 | RBBP | 1970 | 2013 |  |  |  |  |
| Woodpigeon | *Columba palumbus* | 43 | BoCC | 1970 | 2013 |  |  |  |  |
| Wren | *Troglodytes troglodytes* | 43 | BoCC | 1970 | 2013 |  |  |  |  |
| Wryneck | *Jynx torquilla* | 43 | RBBP | 1970 | 2013 | 1978 | 21 | 2001 | 1 |
| Yellow Wagtail | *Motacilla flava* | 43 | BoCC | 1970 | 2013 | 1978 | 1.107 | 2009 | 0.258 |
| Yellowhammer | *Emberiza citrinella* | 43 | BoCC | 1970 | 2013 | 1975 | 0.931 | 2013 | 0.447 |
